# The Use of Mixed Effects Models for Obtaining Low-Cost Ecosystem Carbon Stock Estimates in Mangroves of the Asia-Pacific

**DOI:** 10.1371/journal.pone.0169096

**Published:** 2017-01-09

**Authors:** Jacob J. Bukoski, Jeremy S. Broadhead, Daniel C. Donato, Daniel Murdiyarso, Timothy G. Gregoire

**Affiliations:** 1 School of Forestry and Environmental Studies, Yale University, New Haven, CT, United States of America; 2 Food and Agricultural Organization, United Nations, Bangkok, Thailand; 3 Spatial Informatics Group, Pleasanton, CA, United States of America; 4 Department of Natural Resources, Washington State, Olympia, WA, United States of America; 5 Center for International Forestry Research (CIFOR), Bogor, Indonesia; 6 Department of Geophysics and Meteorology, Bogor Agricultural University, Bogor, Indonesia; Griffith University, AUSTRALIA

## Abstract

Mangroves provide extensive ecosystem services that support local livelihoods and international environmental goals, including coastal protection, biodiversity conservation and the sequestration of carbon (C). While voluntary C market projects seeking to preserve and enhance forest C stocks offer a potential means of generating finance for mangrove conservation, their implementation faces barriers due to the high costs of quantifying C stocks through field inventories. To streamline C quantification in mangrove conservation projects, we develop predictive models for (i) biomass-based C stocks, and (ii) soil-based C stocks for the mangroves of the Asia-Pacific. We compile datasets of mangrove biomass C (197 observations from 48 sites) and soil organic C (99 observations from 27 sites) to parameterize the predictive models, and use linear mixed effect models to model the expected C as a function of stand attributes. The most parsimonious biomass model predicts total biomass C stocks as a function of both basal area and the interaction between latitude and basal area, whereas the most parsimonious soil C model predicts soil C stocks as a function of the logarithmic transformations of both latitude and basal area. Random effects are specified by site for both models, which are found to explain a substantial proportion of variance within the estimation datasets and indicate significant heterogeneity across-sites within the region. The root mean square error (RMSE) of the biomass C model is approximated at 24.6 Mg/ha (18.4% of mean biomass C in the dataset), whereas the RMSE of the soil C model is estimated at 4.9 mg C/cm^3^ (14.1% of mean soil C). The results point to a need for standardization of forest metrics to facilitate meta-analyses, as well as provide important considerations for refining ecosystem C stock models in mangroves.

## Introduction

Recent studies have identified mangrove forests as carbon (C) rich ecosystems, with substantial stocks of C found in both their living tree biomass and soils [1–4]. C-rich ecosystems carry special value under existing international efforts to reduce greenhouse gas emissions, as conversion of these lands to other uses results in large CO_2_ emissions [5, 6]. Mangroves also provide a range of benefits beyond the sequestration of C, such as the support of local livelihoods, biodiversity conservation, water filtration, coastal protection, and nutrient cycling [1, 7–11]. Given both their C-rich nature as well as their socio-economic importance, mangrove conservation is increasingly emphasized as an evident opportunity for joint climate change mitigation (reduction of greenhouse gas emissions) and adaptation (increasing resiliency to climate change induced stressors) projects [12, 13].

Nevertheless, mangroves remain highly threatened from both direct and indirect anthropogenic pressures despite their established importance [8, 14–16]. Mangroves have historically been under direct pressure from aquaculture (e.g., shrimp farming), which is often deemed an economically more productive activity than alternative, less-destructive land use options [4, 17]. Recent research has shown that expansion of oil palm plantations, rice paddies, and coastal urban development have also been substantial but over-looked land-use competitors for Southeast Asian mangroves from 2000–2012 [18]. Furthermore, mangroves are projected to be adversely impacted by rising mean sea levels, which may alter hydrological regimes, shift species compositions, or drown entire forest stands [4, 19, 20].

The substantial C stocks of mangroves suggest that payments for their conservation through C forestry mechanisms (CFMs) could be effective in simultaneously mitigating atmospheric CO_2_ levels, securing non-C ecosystem services, and providing benefits to local livelihoods [1, 21–23]. However, CFM initiatives currently experience a range of implementation challenges, including the difficulty of quantifying forest C stocks through measuring, reporting, and verifying (MRV) activities and the scale of their associated costs [24–26]. Soft soils, complex aboveground root systems, and tidal windows constrain accessibility in mangroves, which render MRV activities particularly difficult to implement and often necessitate the involvement of specialists. These challenges are especially likely to impose economic limits on the inclusion of less extensive, yet ecologically and socioeconomically important, patches of mangroves in CFM projects [25]. As a response to the challenges of CFM MRV activities, practitioners are now seeking more efficient means of obtaining robust and transparent quantification of C stocks in both mangroves and tropical forests more generally [27, 28].

The use of statistical regression models that correlate well with C stocks is one potential means of reducing the cost of MRV activities [29]. Predictive models of mangrove biomass (a proxy for live C stocks) have been examined, although infrequently, in past decades. One of the first models examined the relationship between mangrove tree height and soil salinity in Puerto Rico [30]. Other attempts have correlated mangrove biomass to latitudinal distance from the equator, which is understood to have a negative impact on mangrove tree growth [31]. More recent attempts have produced global maps of both mangrove biomass C and soil organic C (SOC) from climatic datasets and regional indicator variables [32, 33]. These models primarily predict at the global scale, sacrificing accuracy for widespread applicability, and thus there may be room for improvement in predictive power at the micro- or meso-scale by incorporating site-level parameters such as forest structure or species composition. Predicting C stocks at the site-scale is expected to help operationalize CFMs and other mangrove conservation efforts that require valuation of ecosystem services.

Mangroves exhibit spatial variation in both the presence and abundance of plant species, as well as in tree size and total biomass across intertidal zones [34]. This phenenomenon is termed “zonation,” and likely has significant implications for C stocks [29, 35–37]. The dominant genera of mangrove trees present in a given stand dictate tree morphology and wood density, which are likely to be efficient predictors of biomass C stocks [2]. Additionally, the dominant genera of a stand may be indicative of tidal position, which may dictate the rate of organic matter decomposition in soils. Recent studies have found evidence that species composition and SOC stocks covary with intertidal zone [29, 37]. Similarly, other research that investigated spatial variation in soil CO_2_ fluxes, an indicator of organic matter decomposition, found significant variation with intertidal zone [38]. Recent studies in terrestrial tropical biomes more generally have identified the presence of “hyperdominant” species (i.e. species that comprise less than 2% of the total species count but more than 50% of biomass) as habitat specialists, which may parallel the phenomenon of zonation in mangroves [39–41]. Although site-specific parameters such as dominant genera or forest structure are challenging to incorporate in models that are global in scope, they are feasible in models intended to be implemented at the micro- or meso-scale.

This paper presents predictive models of biomass-based and soil-based C stocks in mangroves that employ latitude, forest structure and species composition parameters as potential predictors. We hypothesize that by including forest structure and composition parameters, more precise estimates of site-specific mangrove C can be achieved. Furthermore, it is not always clear whether other predictive models of mangrove C stocks within the academic literature account for spatial correlation across sites, which can inflate the model’s degrees of freedoms and induce narrower error bounds than is appropriate. By employing a linear mixed effects model, we can account for spatial autocorrelation within the dataset and avoid the risk of inflated estimates of model precision, as well as make inferences on how variation in mangrove C stock per unit area estimates vary both within and across sites. The models presented in this paper comprise one output of the “Income for coastal communities for mangrove protection project”, which is implemented by the Mangroves for the Future regional programme in partnership with the United Nations Food and Agriculture Organization and the United States Agency for International Development’s Lowering Emissions in Asia’s Forests program.

## Methods

### Literature search and data quality

To parameterize the C stock model, we conducted an extensive review of major academic databases. The terms “mangrove” plus “biomass,” “C stocks,” “ecosystem C” and “C sequestration” were searched for within Google Scholar, Web of Science, ScienceDirect, and Springer. From there, we identified additional studies via those referenced within relevant articles, as well as through colleagues in the Asia-Pacific region.

As literature on mangrove C stocks reports a range of forest structure parameters and sampling methods, we made a judicious effort to compile subsets of data for studies that reported comparable stand-level estimates for each of (i) height, (ii) mean stem diameter at breast height (1.37 m; DBH), (iii) mean stem density, and (iv) mean basal area. For studies that reported two of the last three structure parameters, the third was estimated algebraically (e.g., computing basal area as a function of mean stand diameter and mean stem density). To limit the variation across aggregate forest structure parameter estimates, only studies that employ a sampling cut-off of stem diameters 5 cm or less were included in the dataset. The augmented variance from small fluctuations in sampling design (i.e. a cutoff of 2.5 cm versus 5 cm) is tolerated and ultimately reflected in the model fit.

### Field sampling for additional data

In addition to legacy data from the literature, we sampled ecosystem C stocks at two sites in south Thailand and three sites in north Vietnam from May to August of 2015. Research permissions were obtained from the Department of Agriculture and Rural Development in Vietnam, and the Royal Forest Department of Thailand. All sampling at sites with planted mangroves were publicly owned, and thus no additional permissions from private owners were required.

The field sampling methods generally follow those outlined by Kauffman and Donato [42]. We randomly located transects, each consisting of five to six circular subplots of 7 m radius, oriented perpendicularly to the coastline. All stem diameters (DBH ≥ 5 cm) were measured within the subplot, whereas saplings (DBH < 5 cm and height ≥ 1.37 m) and seedlings (height < 1.37 m) were measured within a nested 2 m radius subplot. We converted tree diameters to kg of dry-weight biomass and subsequently kg of C via species-specific allometric equations, or a general equation with species-specific wood densities when species-specific equations were not available. All stem measurements were taken at the appropriate species-specific stem positions following predesignated allometric equations. Additionally, soil cores up to 2 m depth were taken at two randomly selected subplots along each transect. Subsamples from each of the 0–15, 15–30, 30–50, 50–100, and 100–200 cm depth intervals were analyzed for bulk density and percent organic C via dry combustion. Additional details on the sampling design, soil analysis, and allometric equations are provided in the S1 File of the Supporting Information.

### Development of the predictive models

For the development of the predictive models, we specify two response variables. For the biomass C model, the response variable is biomass C in Mg C/ha whereas for the SOC model, the response variable is SOC in g/cm^3^. The rationale behind predicting SOC per cm^3^ rather than per ha is that soil depth in mangroves is believed to be highly variable and would likely break down predictive relationships between SOC stocks and forest structure or species composition parameters. If accurate estimates of SOC in g/cm^3^ can be obtained, predicted SOC values can be easily coupled with inexpensive field soil depth measurements to obtain site-wide estimates of SOC.

Mangrove C stock data is sparse within the published literature. Commonly, these data arise from disparate sampling methods and reporting of forest structure parameters. As such, a multiple-linear regression model that employs mixed effects is most appropriate as it allows for the preservation of spatially-correlated observations, and thus the maximum dataset size. Mixed effects models are useful in their specification of both “fixed” as well as “random” effects. Although fixed and random effects have varied definitions within the academic literature, we refer to them here following Kreft and Leeuw’s [43] definitions: fixed effects, which explain variation in the dataset expected to hold across the entire population of data, versus random effects, which explain variation unique to specific groupings (i.e. site) of the data. Mixed effects models are increasingly used as a means of dealing with simple spatial pseudoreplication in the academic literature, particularly within island biogeography studies [44–46]. By specifying a mixed effects model to predict mangrove C stocks, we can preserve clustered observations from single sites and avoid discarding of valuable data.

We consider fixed effects for four forest structure parameters, namely forest height, mean DBH, mean stem density, and mean basal area, as well as the dominant genera of the plot, the geomorphic condition (i.e. marine vs estuarine) and latitude, whereas we specify random effects by site. It is understood that C stocks will be spatially correlated within sites, as common factors such as annual temperatures, rainfall, soil quality, tidal regime, or anthropogenic disturbance patterns will impact site productivity. We assume that the effects of forest structure (e.g., DBH or basal area) are constant across sites, and thus we only examine random intercepts (i.e. no random slopes) for site. Furthermore, by specifying fixed effects interactions between latitude and structural parameters, we can account for the influence that any latitudinal changes in rainfall and insolation may have on forest structure without specifying random slope effects for these predictors. The specification of slope coefficients as fixed effects ensures that the major predictive effects will be applicable across all mangroves within the broader Asia-Pacific region. The most parsimonious model is achieved by elimination of non-significant covariates using chi-squared testing at the 5% significance level (p = 0.05), and is specified as

$$\begin{array}{c} Y_{i}=\beta_{i}*X_{i}+b_{i}+\epsilon_{i} \end{array}$$ (1)

where *Y*_*i*_ is the dependent variable for the i^th^ observation, *X*_*i*_ is a row vector of *f* fixed predictor variable values including an intercept, *β*_*i*_ is a vector of fixed effect coefficients, *b*_*i*_ is a vector of random effect intercepts, and *ϵ*_*i*_ is the random deviation from expectation. To examine the variation explained by fixed versus random effects, we employ marginal and conditional R^2^ values via the ‘MuMin’ package of Program R [47–49]. Marginal R^2^ values are representative of the percent variation explained by the fixed effects, whereas the conditional R^2^ values are representative of the percent variation explained by both the fixed and random effects [47]. Both the biomass and SOC models are parameterized using all predictor variables initially, followed by one-by-one elimination of non-significant variables (p >0.05) and starting with the predictor of least significance (highest p-value) first. After elimination of a single non-significant predictor variable, the model was reparameterized and the process repeated until all predictor variables were significant at the 5% level. Additionally, logarithmic transformations for all predictor variables in both models are examined, though a preference is given for untransformed predictors. We fit all models using maximum likelihood estimation for elimination of non-significant fixed effects parameters, whereas we derive the final model parameters using restricted maximum likelihood estimation. All modeling is performed with the ‘lme’ function of the ‘nlme’ package of R [50].

### Model validation

The presence of random effects in mixed effects models creates complications for validation procedures. For true out-of-sample validation, entire groups (i.e. sites) must be withheld during model validation, or else the model runs the risk of being “trained” on observations from the same sites as observations included in the validation set. Thus, the model is validated by both goodness of fit measures and randomly selecting and withholding approximately 15% of the sites for out-of-sample validation. The limitations of data availability necessitate that 15% as opposed to the conventional one-third of data is withheld for validation procedures. Following validation, the model is re-parameterized with the full dataset to obtain as statistically robust a model as possible. By examining shifts in the model coefficients obtained from parameterization with the full dataset versus the model coefficients obtained during the validation procedures (i.e. a reduced estimation dataset), we can assess the reliability of the parameters for predictive purposes.

## Results

### Compiled literature dataset

**Fig 1:**
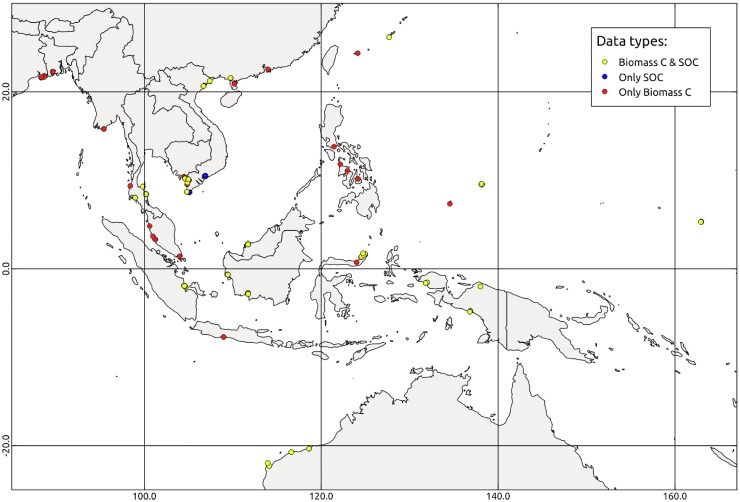
Geographic distribution of data observations. The mapped points represent sites from which plot specific estimates of either biomass C or SOC stocks exist. The administrative boundaries of the countries shown in Fig 1 are adapted from the Natural Earth free open map data.

We retrieved plot-specific dry-weight biomass or dry-weight biomass C including both above- and belowground stocks from a total of 197 observations from 48 sites. Of these observations, 136 observations reported mean DBH, 66 reported mean height, 181 contained estimates of basal area per ha, and 180 reported stem density. Studies reporting SOC estimates were considerably fewer. For SOC, a total of eight studies reported 99 observations from 27 sites (including the empirical measurements of this study). All studies reported the dominant genus of tree present in the plot. The geographic distribution of the biomass C observations is shown in Fig 1. A full accounting of the studies employed in the model fitting, as well as the forest structure parameters reported, are given in the S2 File of the Supporting Information.

### Biomass C model

The most parsimonious biomass C model included fixed effects for both basal area and the interaction between basal area and latitude, as well as random effects for site. The fixed effects specifications for mean DBH, mean canopy height, mean stem density, and dominant genera of tree were not significant at the 95% level, and thus were removed from the model. Additionally, the fixed effect for latitude was not significant following inclusion of the basal area and latitude interaction, and thus it also was removed from the final model. Transformations were not applied to the response variable to avoid correction of back-transformation bias.

The model was validated by withholding all observations from seven sites randomly selected from the parameterizing dataset (i.e. 15% of all sites), and comparing the observed versus predicted values of biomass C stocks for these sites. The validation procedure was run 250 times to examine the distributions of the derived model coefficients and the root mean square error (RMSE). Given that the data are unbalanced (i.e. unequal numbers of observations across sites), the total number of observations withheld during the validation runs varied depending on which sites were randomly selected into the validation set. The average number of observations withheld when randomly selecting seven sites was 29, or approximately 18% of the full dataset. The RMSE between the observed and predicted values of Mg C/ha was 37.7 Mg/ha, approximately 28.2% of the average Mg C/ha value across all sites (133.8 Mg C/ha). However, we note that this value is without the inclusion of random intercepts. The mean values and standard deviations for the number of observations withheld, the model coefficients, and the RMSE are displayed in Table 1.

**Table 1:** Results of the biomass model validation procedures.

	Mean value	Standard deviation
Observations withheld	29	8
Intercept; (*β*_0_)	-8.52	3.00
Basal area; (*β*_1_)	5.75	0.20
Basal.area:Latitude; (*β*_2_)	-0.13	0.02
RMSE (Mg C/ha)	35.94	8.30

**Fig 2:**
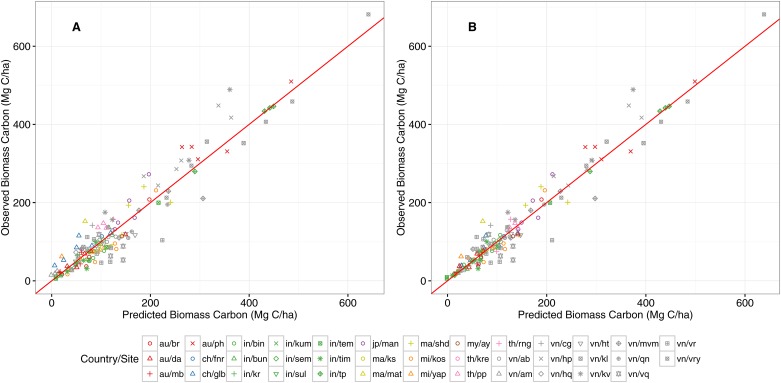
Observed versus predicted plots for the biomass C model. Panel A corresponds to estimation via the fixed effects parameters only, whereas panel B corresponds to inclusion of the random effects. The plotting symbols are colored by country and the symbols correspond to site within country. The key to the country/site codes is provided in S3 File. The red line represents a one-to-one “perfect fit” around which the data should aggregate.

The statistics of the model estimated via the full dataset are displayed in Table 2. The best linear unbiased predictors (BLUPs) for the random site effects are provided in the S3 File of the Supporting Information. Following the inclusion of the BLUPs, the RMSE for the difference between the observed and predicted values is yielded at 24.6 Mg/ha, or 18.4% of the average Mg C/ha value across all sites. Observed versus predicted plots for the model estimates based on fixed effects only and both fixed and random effects are shown in Fig 2.

**Table 2:** The statistics of the model estimated via the full biomass C dataset.

Term	Covariate	Value	Std. Error	Deg. Freedom	p-value
*β*_0_	Intercept	-8.43	3.32	118	0.012
*β*_1_	Basal area	5.76	0.10	118	<0.001
*β*_2_	Basal area:Latitude interaction	-0.13	0.01	118	<0.001

### SOC model

The most parsimonious SOC model included fixed effects for the logarithmic transformations of both latitude and basal area, as well as random effects for site. An investigation of the marginal R^2^ (49.1%) and conditional R^2^ (89.9%) values reveals that a substantial proportion of the model’s explanatory power is housed within the specification of random effects. None of the other structural predictors nor dominant vegetation genus within the plot (eight levels, specified for *Aegiceras, Avicennia, Bruguiera, Ceriops, Excoecaria, Kandelia, Rhizophora,* and *Sonneratia*) were found to be significant predictors at the 95% level. The observed versus predicted plots following prediction via both (i) fixed effects only and (ii) mixed effects reveal the power of including the random effect BLUPs for site, as the variation around the one-to-one identity line is considerably greater for the fixed effects only prediction.

The model was validated by withholding all observations from four sites (15% of the SOC dataset) for each paramaterization/validation run. Similar to that of the biomass model, we ran the validation procedure a total of 250 times (randomly selecting the four sites for withholding each time) to examine the distributions of the derived model coefficients and RMSE. The mean number of observations withheld was 19 observations (from a total dataset size of 99 observations), or approximately 20% of the full dataset. The RMSE between the observed and predicted SOC values of the validation procedure was 13.5 mg/cm^3^, or approximately 38.0% of the mean SOC value across the full dataset. The full results of the SOC model validation procedure are given in Table 3. It is important to note that the validation procedure has used predicted values from only the fixed effects component of the model, and thus for sites in which the random effects BLUPs could be estimated, we can assume the uncertainty levels of the SOC model will decrease.

**Table 3:** Results of the SOC model validation procedures.

	Mean value	Standard deviation
Observations withheld	19	11
Intercept; (*β*_0_)	38.71	2.89
Latitude; (*β*_1_)	-11.24	0.91
Basal.area; (*β*_2_)	4.53	0.42
RMSE (mg C/cm^3^)	13.47	3.28

**Fig 3:**
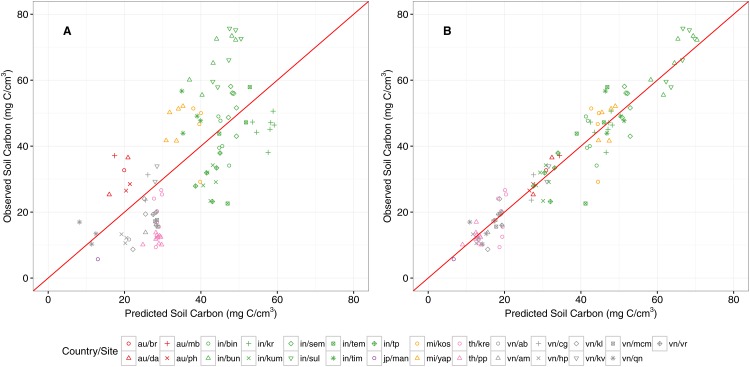
Observed versus predicted plots for the soil organic C model. Panel A corresponds to estimation via the fixed effects parameters only, whereas panel B corresponds to inclusion of the random effects. The plotting symbols are colored by country, whereas the symbols correspond to site within country. The key to the country/site codes is provided in S3 File. The red line represents a one-to-one “perfect fit” around which the data should aggregate.

Following the model validation procedure, we fit the model on the full dataset. The RMSE value of the fixed effects only model is estimated at 13.4 mg C/cm^3^, or approximately 38.6% of the mean SOC value (34.6 mg C/cm^3^) across the dataset, whereas the RMSE value of the mixed effects model is estimated at 4.89 mg C/m^3^ (approximately 14.1% of the mean SOC value). The final model parameters are provided in Table 4, and the observed vs. predicted plots following model estimation with both fixed effects only and mixed effects are shown in Fig 3.

**Table 4:** The statistics of the model estimated via the full biomass C dataset.

Term	Covariate	Value	Std. Error	Deg. Freedom	p-value
*β*_0_	Intercept	38.62	7.11	73	<0.001
*β*_1_	log(Latitude)	-11.31	2.31	73	<0.001
*β*_2_	log(Basal area)	4.48	1.45	73	0.003

## Discussion

The predictive model of mangrove biomass C stocks performs well. The higher predictive capability of basal area over the other forest structure parameters is primarily a result of accounting for both mean tree diameter as well as mean stem density. For the same reasons, basal area may better account for forest degradation induced from selective logging whereas other forest structure parameters (e.g., height or mean stem diameter) and ecological variables (e.g., climatic conditions) do not. As low temperatures are known to stunt mangrove growth, the interaction between latitude and basal area does well to correct the model at sites of higher latitude, which may exhibit lesser standing volumes of mangrove biomass for a given basal area per ha estimate [31]. The finding that basal area is a significant predictor of mangrove biomass C has important implications for estimating biomass C, as basal area estimates can be obtained via horizontal point sampling (e.g., the use of angle gauges, relascopes, or similar instruments). Horizontal point sampling requires substantially less time and energy in the field than conventional measurements (i.e., establishment of plots and measurements of stem diameters), and may help streamline biomass C quantification in mangroves. The value of basal area as a streamlined predictor of stand-level biomass C stock estimates has been noted by others [27].

The predictive power of the SOC model is greatly enhanced by including the random effect BLUPs specified by site. This indicates that for the dataset we compiled from the academic literature (i.e. plot specific values of location, dominant genus of tree, geomorphic condition, mean DBH, mean canopy height, basal area and stem density), a substantial proportion of the variation within the dataset is housed within site-to-site variation. Although there was a significant correlation between the logarithmic transformations of both latitude and mean standing basal area, the model fit is greatly enhanced by accounting for site-to-site differences. This indicates that factors unique to each site substantially influence the accretion of SOC stocks. For example, surrounding upland land uses that drain through the site may potentially dictate the amount of suspended organic material that passes through mangroves, and may influence SOC accretion rates. Further research on mangrove SOC stocks and the adjacent land uses from a remote sensing perspective could help elucidate the degree to which these relationships do or do not exist.

Although our hypothesis that SOC would correlate well with dominant tree species of vegetation was not substantiated by our modeling efforts, there are other considerations that may have eroded the correlation between the two variables. First and foremost, mangrove stands are often characterized by mixtures of tree species rather than a single, indisputably dominant genus. Although certain species may be indicative of tidal position, such distinctions are rarely ever clearly defined. Second, a lack of consistency in how the dominant genus of a plot is identified within the academic literature may have further degraded the correlation between the two variables. Authors may estimate the dominant genus of a plot visually (e.g., canopy dominants) or through measures that range in complexity from univariate measures (e.g., percent plot basal area) to relative importance indices based on multiple considerations (e.g., density, frequency, and canopy coverage).

Similarly, geomorphic condition as a predictive variable was likely non-significant in our analyses due to its categorical nature and lack of quantitative basis that more closely describes ecological processes. Furthermore, the differences in SOC seen between different geomorphological positions (i.e., marine vs. estuarine) are often presented through absolute stocks, and significant differences in soil depth between marine and estuarine mangroves is a key factor. In our modeling effort, we have removed the influence of soil depth which may further account for the lack of correlation between geomorphic condition and mangrove SOC. Others have noted the point that geomorphic condition is often qualitatively defined, and have recently sought to institute ecologically-derived classifications of geomorphic condition for mangroves globally [51]. It is possible that other indicators of tidal position (e.g., Euclidean distance from shoreline or soil salinity) would correlate better to SOC, as spatial variation in vegetation and ecosystem C stocks has been noted well within the literature [29, 36, 37, 52]. However, distance from shoreline and soil salinity are not consistently reported within the academic literature, and thus we did not incorporate them into the models of this study.

While the mixed effects model structure allows for compilation of a robust dataset from various studies, it also presents unique challenges for model application. Prediction of mangrove biomass C via only fixed effects has higher RMSE (i.e. higher uncertainty), but is applicable throughout the region regardless of site. Conversely, prediction of mangrove biomass C via both fixed and random effects has a lower RMSE, but is restricted to the sites for which the BLUPs of the random effects have been estimated. For predicting stocks at sites of interest in close proximity to those for which random intercepts exist, inclusion of the random intercepts may be appropriate to obtain more precise estimates; however, it is important to give judicious consideration of whether the ecological and anthropogenic characteristics of the two ecosystems are sufficiently similar to justify doing so. The random effect BLUPs can be estimated for new sites via a full biomass inventory of a single plot (i.e. measurement of stem diameters and conversion to biomass volume via allometric equations), and re-estimation of the model parameters.

### Model vs. site-level uncertainty

It is important to note here that that the RMSE values provided in this study are not direct representations of final uncertainty surrounding an estimate of C stocks at the site level, but are estimates of model uncertainty analogous to the error bounds surrounding an allometric equation. Variation in C stock estimates at the site level is a function of i) model uncertainty, ii) measurement uncertainty, and iii) sampling uncertainty, and thus the RMSE values reported here are only one component of uncertainty at the site level. Although studies have called for the propagation and reporting of all three uncertainty pools, C stock appraisals most commonly report only the uncertainty from sampling design (i.e. plot to plot variation). The inclusion of other uncertainty pools requires additional computations through error propagation, and is thus rarely included in uncertainty surrounding site-level C stock estimates. Furthermore, estimation of the measurement uncertainty pool requires multiple measurements of single plots, which may be deemed an inefficient use of resources.

Although some have noted that model uncertainty is the biggest contributor to site level error bounds [53], other studies have concluded that sampling variation is the largest contributor to site-level C stock estimate error bounds, particularly for sites with high levels of variation in species composition or forest structure [54–56]. The use of basal area as a model input allows for more rapid sampling of plot level data (horizontal point sampling vs. conventional DBH sampling), which will allow for the sampling of a greater number of plots per given sampling effort and thus likely reduce the levels of sampling uncertainty. Others have noted that devoting resources towards sampling of more numerous points is particularly useful for monitoring changes in forest C stocks, due to a more comprehensive accounting of spatial heterogeneity [56, 57]. Monitoring changes in forest C stocks is of particular importance for CFM projects, as the additional sequestration of C relative to a baseline value is key to project success.

A hybrid method of field sampling in which conventional measurements of a small subset of plots (e.g., measurements of DBH and conversion to biomass C via allometric equations or collection of soil cores) is conducted and followed by conversion of basal area measurements to C stock values at numerous other plots via the models presented in this study may be three-fold valuable for reducing site-level uncertainty. First, the conventionally-sampled plots could be used for reparameterization of the model coefficients and thus the random effect BLUPs for out-of-sample sites could be obtained to reduce model uncertainty. Secondly, conventionally-sampled plot estimates of stem diameters could be converted to basal area estimates and used to calibrate measurements of basal area, ensuring against systematic measurement error in the basal area estimates. Finally, the streamlined nature of collecting basal area estimates via horizontal point sampling (i.e., use of an angle-gauge, relascope, or similar instrument) expedites repeated measurement of single plots, and can facilitate the inclusion of measurement uncertainty in site-level error bounds. Furthermore, soil depth measurements could be collected cheaply in conjunction with the basal area measurements, allowing for more comprehensive estimation of SOC stocks. Although SOC is expected to vary across soil profile depths, it has been shown that often-times this variation is non-significant [4]. For users of the model concerned with variation in SOC along the soil profile depth, applying the model estimates to just the top 1 m of soil is appropriate.

## Conclusion

As a potential means of streamlined C stock estimation in mangroves, we developed two predictive models for each of biomass-based C stock and soil-based C stocks in the Asia-Pacific. To maximize the preservation of data, we employed a multiple-linear regression model with mixed effects that allows spatial correlation of individual observations. The final model forms reveal that use of basal area and latitude are significant predictors of mangrove C stocks, but accounting for site-specific factors substantially improves our model fits. Thus, a hybrid sampling approach in which a reduced number of conventionally-sampled plots paired with rapid estimates of basal area at more numerous plots may be an efficient use of the models presented in this study. Although our modeling efforts did not substantiate our hypothesis that SOC stocks would correlate well with zonation in vegetation, this may be partially due to a lack of consistent reporting of data within the academic literature. Examining other measures of tidal position, such as Euclidean distance to shoreline, may prove more fruitful but may also encounter challenges due to the dynamic, imprecise definition of coastlines.

## Supporting Information

S1 File Additional details of field sampling. The additional details of the field sampling for collection of ecosystem C stock estimates are contained in this file.
(PDF)

S2 File Modeling dataset. The full dataset and references for estimation of the model parameters are given in this file.
(PDF)

S3 File Random effect intercept values. The best linear unbiased predictors (BLUPs) of the random effects are provided in this file.
(PDF)

S4 File R script for statistical analyses. An R script providing the details of the statistical analyses is provided in this file. The script is provided in hopes of improving transparency and the application of our models to future datasets.
(R) Click here for additional data file.
