# Supplementary material for: The Use of Mixed Effects Models for Obtaining Low-Cost Ecosystem Carbon Stock Estimates in Mangroves of the Asia-Pacific
Source: PLoS One. 2017 Jan 9;12(1):e0169096. doi: 10.1371/journal.pone.0169096 (PMC5222395; doi:10.1371/journal.pone.0169096)
Supplement: S1 File — The additional details of the field sampling for collection of ecosystem C stock estimates are contained in this file. (PDF) [file pone.0169096.s001.pdf]

Supporting Information 1
Additional details of field sampling

The following supplementary information file provides details of the field sampling methodology used to collect additional carbon stock data for development of the predictive models, as well as the full results of the compiled dataset that emerged from the academic literature.

1 Field sampling methods

The field sampling methods are described for each of five sites visited. Two of the sites were located in south Thailand (the Krabi River Estuary of Krabi province, and Pak Panang of Nakorn Si Thammarat province), while the remaining three were located in north Vietnam (Giao Thuy of Nam Dinh province, Quang Vinh of Hai Phong province, and Dong Rui of Quang Ninh province). The mangrove forests in Krabi, Nakorn Si Thammarat, and Quang Ninh were primary or secondary forests with diverse species compositions, whereas the mangrove forests of Nam Dinh and Hai Phong were monospecific plantations.

1.1 Sampling design

A simple random sample was taken from each site to quantify each of the above- and belowground biomass pools. An initial transect starting point was located randomly via geographic information system (GIS) software within the forest, and the closest shoreline to the point (excluding minor tributaries) was identified. The closest point on the shoreline and the randomly located point were used as the two ends of the transect, and six subplots were laid out at 25m intervals [1]. The number of transects for each site was determined by a desired margin of error ($t = 1.645$) of approximately 30% for the aboveground biomass components. Basic statistics were run after each day in the field, and additional transects were laid to reduce uncertainty to desired levels. At Krabi and Pak Panang, each additional transect was located randomly within the forest area. For Giao Thuy, Quang Vinh and Dong Rui, each transect was laid in parallel approximately 100 meters from the pre-existing transect from the day before due to resource and site access limitations. As the sampling is designed to obtain stand-level estimates in Mg C/ha for model parameterization rather than total site-level estimates, the absence of randomly locating each transect within the north Vietnamese sites is deemed acceptable.

Table 1: **Qualitative description of the five mangrove forests sampled**

Site	Latitude	Longitude	Forest type	Dominant species	Stature	Type
Krabi	8.04°N	98.9°E	Secondary	Mixture	Mature	Estuarine
Pak Panang	8.50°N	100.2°E	Primary/secondary	Mixture	Mature	Marine
Giao Thuy	20.0°N	106.0°E	Plantation	*Kandelia obovata*	Young	Marine
Quang Vinh	20.5°N	106.6°E	Plantation	*Sonneratia caseolaris*	Mature	Marine
Dong Rui	21.2°N	107.4°E	Primary	Mixture	Stunted	Marine

1.2 Field methods

For the three mature mangrove forests (Krabi, Pak Panang, and Quang Vinh), the methods recommended by Kauffman and Donato for sampling biomass were followed [1]. The morphologies of mangroves present complications for measuring stem diameter, as often-times no true stem exists at breast height. As such, the appropriate position at which stem diameters should be measured must be identified by the species- and region-specific allometric equations to be used before entering the field. The diameters of all stems greater than 5 cm at the appropriate height were measured throughout each subplot of 7 m radius, whereas the diameters of saplings (denoted by a diameter < 5 cm at the appropriate height) were recorded within the 2 m nested plot. All diameters were converted to biomass via their species-specific allometric equations, which are described in detail in the Allometry section below. Seedlings were denoted as stems less than 1.37 m in height, and were simply counted within each 2 m nested plot. Understory vegetation is sparse in mangroves, and is often a negligible carbon pool at the ecosystem level. All biomass estimates are converted to carbon via a factor of 0.48 for aboveground biomass, and a factor of 0.39 for belowground biomass as recommended by Kauffman and Donato [1].

The average stem diameter of the mangrove forests in Quang Vinh and Dong Rui were less than 5 cm, and thus the cut-offs for inclusion of stems vs. saplings vs. seedlings had to be adjusted. The diameters and heights of all stems taller than 1.37 m within the 7 m radius plot were measured (denoted as trees), whereas all stems within the 2 m nested plot less than 1.37 m in height (denoted saplings) were simply counted. Typically, mangroves of this structure are destructively sampled to obtain biomass estimates, which is both expensive and environmentally damaging. Instead of employing destructive sampling methods, a relationship between the biomass and height of stems was developed, and a simple ratio was applied to obtain a biomass value for a sapling of average height (i.e. 0.65 m). The corresponding average value of biomass per sapling was then multiplied by the number of saplings contained within each plot to obtain the biomass pool for saplings.

Soil samples were taken at two of six subplots for each transect in Quang Vinh and Dong Rui, and from one subplot along the transect in Giao Thuy. An open-face peat auger of 1 m length was used to collect

an initial soil core, from which 4 soil samples of 3-5 cm were taken from each of the 0-15, 15-30, 30-50, and 50-100 cm soil core intervals. If an extracted soil core exhibited too much compaction or sloughing of soil, the core was discarded and an additional one was taken. Following the first core, a second core was taken from a 1-2 meter soil depth and a fifth 3-5 cm sample was taken. Soil samples were analyzed for bulk density and percent organic C at either the Faculty of Forestry at Kasetsart University, Thailand, or the Environment Laboratory of the Hanoi University of Natural Resources and Environment, Vietnam. Bulk density was determined as dry weight per unit volume, whereas percent organic C was determined via loss on ignition analysis. Soil organic C was determined for each sample by multiplying percent organic carbon and bulk density values.

1.3 Allometry

The morphology of various mangroves species creates complications for use of allometry, with true stems at breast height (1.37 m) not existing for several species that often dominate stands (e.g., *Rhizophora*) [2]. The diameters of stems were measured at either 1.37 m if a true stem existed, or 30 cm above the highest stilt root if no true stem existed. For multi-stemmed trees, each stem was treated as a distinct individual with an associated share of common biomass (e.g., canopy biomass) defined by its relative diameter [3]. Species-specific allometric equations developed from regions in close proximity to the sampling sites were selected when available, and a general equation that employs species-specific wood-densities used otherwise to estimate volumes of aboveground biomass [4]. Species-specific wood densities were used when available, however if not available for a given genus, all available wood densities for the genus were averaged together [5, 4, 6]. A general equation was used for belowground biomass as belowground biomass studies are sparse within the literature, with the exception of *Rhizophora* sps whose distinct morphologies necessitate special attention. Additionally, *Kandelia candel* was recently classified as *Kandelia obovata* and thus an allometric equation developed for *Kandelia candel* is used [7].

Table 2: **Allometric equations and wood densities employed for the conversion of stem diameter measurements to kg dry-weight of aboveground biomass.**

Species	Density	Allometric equation	D. range (cm)	Reference
Aegiceras corniculatum	0.700	$log(AGB) = 1.496 + 0.465 * log(D^2 * H)$	3.7-36.9	[8]
Avicennia marina	0.650	$log_{10}(AGB) = -0.7506 + 2.2990 * log_{10}(D)$	2.3-13.8	[3]
Bruguiera gymnorrhiza	0.710	$log_{10}(AGB) = -0.7309 + 2.3055 * log_{10}(D)$	2-24	[9]
Bruguiera parviflora	0.760	$log_{10}(AGB) = -0.7045 + 2.5336 * log_{10}(D)$	4-16	[10]
Excoecaria agallocha	0.416	$log(AGB) = 1.0996 * log(D^2) - 0.8572$	2.1-21.6	[11]
Kandelia obovata	0.525	$AGB = 13.3 * (D_{30})^{1.21}$	0.6-14.0	[12]
Lumnitzera racemosa	0.710	$AGB = 0.184 * D^{2.384}$	NA	[13]
Rhizophora apiculata	0.850	$log(AGB) = 2.318 * log(D) - 1.671$	3.5-88	[14]
Rhizophora stylosa	0.840	$log_{10}(AGB) = -0.6564 + 2.4292 * log_{10}(D)$	5.5-20.4	[3]
Avicennia alba	0.587			
Avicennia officinalis	0.605			
Bruguiera cylindrica	0.720			
Bruguiera sexangula	0.740	$AGB = 0.251 * WD * D^{2.46}$	5.0-48.9	[4]
Rhizophora mucronata	0.821			
Sonneratia caseolaris	0.389			
Xylocarpus granatum	0.567			
Xylocarpus moluccensis	0.611			

AGB = Aboveground biomass; log = base-e logarithm; log_{10} = base-10 logarithm; D = stem diameter taken at species-appropriate height (cm); D_{30} = stem diameter taken at 30 cm height (cm); H = height of stem (m); WD = wood density

Table 3: **Belowground biomass allometric equations**

Allometric equations employed for the conversion of stem diameter measurements to kg dry weight of belowground biomass. The species-specific wood densities employed in the Komiyama et al. equation are reported in Table S2.

Species	Allometric equation	D. range (cm)	Reference
Rhizophora apiculata	$log_{10}(BGB) = 1.522 * log_{10}(D) - 1.707$	3.5-77	[14]
Rhizophora stylosa	$log_{10}(BGB) = -0.583 * log_{10}(D)^{1.86}$	3-10	[15]
All other species	$BGB = 0.199 * WD^{0.899 * D^{2.22}}$	5-48.9	[4]

BGB = Belowground biomass;

References

[1] Kauffman JB, Donato D. Protocols for the measurement, monitoring and reporting of structure, biomass and carbon stocks in mangrove forests. Center for International Forestry Research (CIFOR), Bogor, Indonesia; 2012.

[2] Komiyama A, Ong JE, Poungparn S. Allometry, biomass, and productivity of mangrove forests: A review. Aquatic Botany. 2008;89(2):128–137.

[3] Clough B, Dixon P, Dalhaus O. Allometric relationships for estimating biomass in multi-stemmed mangrove trees. Australian Journal of Botany. 1997;45(6):1023–1031.

[4] Komiyama A, Poungparn S, Kato S. Common allometric equations for estimating the tree weight of mangroves. Journal of Tropical Ecology. 2005;21(04):471–477.

[5] Simpson WT. Method to estimate dry-kiln schedules and species groupings: tropical and temperate hardwoods. United States Department of Agriculture; 1996.

[6] Saenger P. Mangrove ecology, silviculture and conservation. Springer Science & Business Media; 2002.

[7] Sheue CR, Liu HY, Yong JW. Kandelia obovata (Rhizophoraceae), a new mangrove species from Eastern Asia. Taxon. 2003; p. 287–294.

[8] Tam N, Wong Y, Lan C, Chen G. Community structure and standing crop biomass of a mangrove forest in Futian Nature Reserve, Shenzhen, China. Hydrobiologia. 1995;295(1-3):193–201.

[9] Clough B, Scott K. Allometric relationships for estimating above-ground biomass in six mangrove species. Forest Ecology and Management. 1989;27(2):117–127.

[10] Hossain M, Othman S, Bujang JS, Kusnan M, Rajagopal S. Allometric relationships for estimating above and below-ground biomass of saplings and trees of Bruguiera parviflora (Wight and Arnold). Malaysian Applied Biology Journal. 2004;33(1):37–45.

[11] Hossain M, Siddique MRH, Saha S, Abdullah SR. Allometric models for biomass, nutrients and carbon stock in Excoecaria agallocha of the Sundarbans, Bangladesh. Wetlands Ecology and Management. 2015;23(4):765–774.

[12] Cue NTK, Ninomiya I. Allometric relations for young Kandelia candel (L.) Blanco plantation in Northern Vietnam. Journal of Biological Sciences. 2007;7(3):539–543.

[13] Kangkuso A, Jamili J, Septiana A, Raya R, Sahidin I, Rianse U, et al. Allometric models and above-ground biomass of Lumnitzera racemosa Willd. forest in Rawa Aopa Watumohai National Park, Southeast Sulawesi, Indonesia. Forest Science and Technology. 2015; p. 1–8.

[14] Ong J, Gong W, Wong C. Allometry and partitioning of the mangrove, Rhizophora apiculata. Forest Ecology and Management. 2004;188(1):395–408.

[15] Comley B, McGuinness K. Above-and below-ground biomass, and allometry, of four common northern Australian mangroves. Australian Journal of Botany. 2005;53(5):431–436.
