# Supplementary material for: The Use of Mixed Effects Models for Obtaining Low-Cost Ecosystem Carbon Stock Estimates in Mangroves of the Asia-Pacific
Source: PLoS One. 2017 Jan 9;12(1):e0169096. doi: 10.1371/journal.pone.0169096 (PMC5222395; doi:10.1371/journal.pone.0169096)
Supplement: S2 File — The full dataset and references for estimation of the model parameters are given in this file. (PDF) [file pone.0169096.s002.pdf]

Supporting Information 2
Full dataset and references for the estimation of the model parameters

Table 1: **The references, carbon stock estimates, and relevant forest structure parameters used for the estimation of the model parameters**

Country	Site	N	AGB/C	BGB/C	SOC	DBH	Height	BA	Stem Density	Reference
Australia	Dampier	2	Y	Y	-	Y	-	-	Y	[1]
Australia	Port Hedland	2	Y	Y	-	Y	-	**	Y	[1]
Australia	Bay of Rest	1	Y	Y	-	Y	-	**	Y	[1]
Australia	Mangrove Bay	1	Y	Y	-	Y	-	**	Y	[1]
Bangladesh	Sundarbans	2	Y	Y	Y	-	-	-	-	[2]
China	Futian Nature Reserve	1	Y	-	-	-	Y	-	-	[3]
China	Futian Nature Reserve	2	Y	Y	Y	Y	-	**	Y	[4]
China	Futian Nature Reserve	1	Y	-	-	Y	Y	Y	Y	[5]
China	Jiulongjiang Estuary	3	Y	-	Y	Y	-	Y	Y	[6]
China	Leizhou Bay	4	Y	Y	-	Y	Y	**	Y	[7]
China	Yingluo Bay	5	Y	Y	Y	-	Y	Y	Y	[8]
F.S. of Micronesia	Kosrae	4	Y	Y	Y	Y*	-	Y*	Y*	[2]
F.S. of Micronesia	Yap	5	Y	Y	Y	Y*	-	Y*	Y*	[2]
India	Sundarbans	5	Y	Y	-	-	-	-	Y	[9]
Indonesia	Awerange	1	Y	-	Y	Y	-	Y	Y	[10]
Indonesia	Bintuni	5	Y	Y	Y	Y*	-	Y*	Y*	[11]

Indonesia	Borneo	5	Y	Y	Y	Y*	-	Y*	Y*	[2]
Indonesia	Bunaken	6	Y	Y	Y	Y*	-	Y*	Y*	[11]
Indonesia	Java	2	Y	Y	Y	-	-	-	-	[2]
Indonesia	Java	1	Y	-	-	**	-	Y	Y	[12]
Indonesia	Kubu Raya	7	Y	Y	Y	Y*	-	Y*	Y*	[11]
Indonesia	Sembilang	6	Y	Y	Y	Y*	-	Y*	Y*	[11]
Indonesia	Sulawesi	6	Y	Y	Y	Y*	-	Y*	Y*	[2]
Indonesia	Tanjung Puting	5	Y	Y	Y	Y*	-	Y*	Y*	[11]
Indonesia	Teminabuan	4	Y	Y	Y	Y*	-	Y*	Y*	[11]
Indonesia	Timika	4	Y	Y	Y	Y*	-	Y*	Y*	[11]
Japan	Manko Wetland	1	Y	Y	-	-	Y	-	Y	[13]
Japan	Manko Wetland	1	Y	Y	-	Y	Y	**	Y	[14]
Japan	Manko Wetland	1	Y	Y	Y	-	Y	-	Y	[15]
Japan	Ishigaki	1	Y	Y	-	-	Y	-	-	[16]
Malaysia	Kuala Selangor	1	Y	Y	-	Y	Y	-	-	[17]
Malaysia	Kuala Selangor	1	Y	Y	-	*	-	*	Y	[18]
Malaysia	Matang Mangrove Reserve	3	Y	-	-	Y	-	**	Y	[19]
Malaysia	Matang Mangrove Reserve	1	Y	Y	-	Y	Y	**	Y	[20]
Malaysia	Sabah	1	Y	-	-	-	-	-	Y	[21]
Malaysia	Sarawak	1	Y	-	-	Y	Y	**	Y	[22]
Malaysia	Sungai Haji Dorani	1	Y	Y	-	*	-	*	Y	[18]

Myanmar	Ayeyarwady Delta	4	Y	Y	-	Y	Y	Y	Y	[23]
Republic of Palau	Palau	1	Y	Y	Y	-	-	-	-	[2]
Philippines	Banacon Island	4	Y	Y	-	Y	Y	-	-	[24]
Philippines	Bugtong-Bato	1	Y	Y	-	-	-	Y	-	[25]
Philippines	Catmon	1	Y	Y	-	-	-	-	-	[26]
Philippines	Poctol	1	Y	Y	-	-	-	-	-	[26]
Philippines	Pedada	1	Y	Y	-	-	-	Y	-	[25]
Singapore	Chek Jawa	10	Y	Y	-	Y	-	-	Y	[27]
Thailand	Krabi River Estuary	7	Y	Y	Y	Y	-	Y	Y	This study
Thailand	Pak Panang	10	Y	Y	Y	Y	-	Y	Y	This study
Thailand	Phuket	1	Y	-	-	-	Y	-	-	[28]
Thailand	Ranong	1	Y	Y	-	Y	-	**	Y	[29]
Thailand	Ranong	2	Y	Y	Y	-	Y	-	-	[30]
Vietnam	An Bien	4	Y	Y	-	Y	Y	Y	Y	[31]
Vietnam	An Minh	5	Y	Y	-	Y	Y	Y	Y	[31]
Vietnam	Ha Tien	4	Y	Y	-	Y	Y	Y	Y	[31]
Vietnam	Hai Phong	3	Y	Y	Y	Y	-	Y	Y	This study
Vietnam	Hon Queo	2	Y	Y	-	Y	Y	Y	Y	[31]
Vietnam	Kien Luong	8	Y	Y	-	Y	Y	Y	Y	[31]
Vietnam	Mui Ca Mao	3	Y	Y	Y	Y	-	*	Y	[32]
Vietnam	Quang Ninh	3	Y	Y	Y	Y	-	Y	Y	This study

Vietnam	Vinh Quang	4	Y	Y	-	Y	Y	Y	Y	[31]
Vietnam	Vam Rang	4	Y	Y	-	Y	Y	Y	Y	[31]
Vietnam	Vam Ray	10	Y	Y	-	Y	Y	Y	Y	[31]

AGB/C = Aboveground biomass/carbon; BGB/C = Belowground biomass/carbon; SOC = Soil organic carbon; N = number of observations (plots); * = Disaggregated forest structure parameters were obtained from the authors; ** = Value was (i) calculated algebraically given other data inputs, or (ii) adjusted to appropriate units

References

[1] Alongi D, Tirendi F, Clough B. Below-ground decomposition of organic matter in forests of the mangroves Rhizophora stylosa and Avicennia marina along the arid coast of Western Australia. Aquatic Botany. 2000;68(2):97–122.

[2] Donato DC, Kauffman JB, Murdiyarso D, Kurnianto S, Stidham M, Kanninen M. Mangroves among the most carbon-rich forests in the tropics. Nature Geoscience. 2011;4(5):293–297.

[3] Li M. Nutrient dynamics of a Futian mangrove forest in Shenzhen, South China. Estuarine, Coastal and Shelf Science. 1997;45(4):463–472.

[4] Luo Z, Sun OJ, Xu H. A comparison of species composition and stand structure between planted and natural mangrove forests in Shenzhen Bay, South China. Journal of Plant Ecology. 2010; p. rtq004.

[5] Tam N, Wong Y, Lan C, Chen G. Community structure and standing crop biomass of a mangrove forest in Futian Nature Reserve, Shenzhen, China. Hydrobiologia. 1995;295(1-3):193–201.

[6] Alongi D, Pfitzner J, Trott L, Tirendi F, Dixon P, Klumpp D. Rapid sediment accumulation and microbial mineralization in forests of the mangrove Kandelia candel in the Jiulongjiang Estuary, China. Estuarine, Coastal and Shelf Science. 2005;63(4):605–618.

[7] Ren H, Chen H, Li Z, Han W. Biomass accumulation and carbon storage of four different aged Sonneratia apetala plantations in Southern China. Plant and Soil. 2010;327(1-2):279–291.

[8] Wang G, Guan D, Peart M, Chen Y, Peng Y. Ecosystem carbon stocks of mangrove forest in Yingluo Bay, Guangdong Province of South China. Forest Ecology and Management. 2013;310:539–546.

[9] Ray R, Ganguly D, Chowdhury C, Dey M, Das S, Dutta M, et al. Carbon sequestration and annual increase of carbon stock in a mangrove forest. Atmospheric Environment. 2011;45(28):5016–5024.

[10] Alongi D, Trott L, Rachmansyah T, Tirendi F, McKinnon A, Undu MC. Growth and development of mangrove forests overlying smothered coral reefs, Sulawesi and Sumatra, Indonesia. Marine Ecology Progress Series. 2008;370:97–109.

[11] Murdiyarso D, Purbopuspito J, Kauffman JB, Warren MW, Sasmito SD, Donato DC, et al. The potential of Indonesian mangrove forests for global climate change mitigation. Nature Climate Change. 2015;5(12):1089–1092.

[12] Sukardjo S, Yamada I. Biomass and productivity of a Rhizophora mucronata Lamarck plantation in Tritih, Central Java, Indonesia. Forest Ecology and Management. 1992;49(3):195–209.

[13] Hoque A, Sharma S, Hagihara A. Above and belowground carbon acquisition of mangrove Kandelia obovata trees in Manko wetland, Okinawa, Japan. International Journal of Environment. 2011;1:7–13.

[14] Khan MNI, Suwa R, Hagihara A. Carbon and nitrogen pools in a mangrove stand of Kandelia obovata (S., L.) Yong: vertical distribution in the soil–vegetation system. Wetlands Ecology and Management. 2007;15(2):141–153.

[15] Khan MNI, Suwa R, Hagihara A. Biomass and aboveground net primary production in a subtropical mangrove stand of Kandelia obovata (S., L.) Yong at Manko Wetland, Okinawa, Japan. Wetlands Ecology and Management. 2009;17(6):585–599.

[16] Suzuki E, Tagawa H. Biomass of a mangrove forest and a sedge marsh on Ishigaki Island, south Japan. Nihon Seitai Gakkaishi. 1983;33(2):231–234.

[17] Hossain M, Siddique MRH, Saha S, Abdullah SR. Allometric models for biomass, nutrients and carbon stock in Excoecaria agallocha of the Sundarbans, Bangladesh. Wetlands Ecology and Management. 2015;23(4):765–774.

[18] Zhila H, Mahmood H, Rozainah M. Biodiversity and biomass of a natural and degraded mangrove forest of Peninsular Malaysia. Environmental Earth Sciences. 2014;71(11):4629–4635.

[19] Alongi D, Sasekumar A, Chong V, Pfitzner J, Trott L, Tirendi F, et al. Sediment accumulation and organic material flux in a managed mangrove ecosystem: estimates of land–ocean–atmosphere exchange in peninsular Malaysia. Marine Geology. 2004;208(2):383–402.

[20] Ong J, Gong W, Clough B. Structure and productivity of a 20-year-old stand of Rhizophora apiculata Bl. mangrove forest. Journal of Biogeography. 1995; p. 417–424.

[21] Faridah-Hanum I, Kudus K, Saari NS, et al. Plant diversity and biomass of Marudu bay mangroves in Malaysia. Pakistan Journal of Botany. 2012;44:151–156.

[22] Arianto CI, Hena A, Kamal M, Gandaseca S. Aboveground biomass production of Rhizophora apiculata Blume in Sarawak mangrove forest. American Journal of Agriculture and Biological Sciences. 2011;6(4):469–474.

[23] Thant YM, Kanzaki M, Ohta S, Than MM. Carbon sequestration by mangrove plantations and a natural regeneration stand in the Ayeyarwady Delta, Myanmar. Tropics. 2012;21(1):1–10.

[24] Camacho LD, Gevaña DT, Carandang AP, Camacho SC, Combalicer EA, Rebugio LL, et al. Tree biomass and carbon stock of a community-managed mangrove forest in Bohol, Philippines. Forest Science and Technology. 2011;7(4):161–167.

[25] Thompson BS, Clubbe CP, Primavera JH, Curnick D, Koldewey HJ. Locally assessing the economic viability of blue carbon: A case study from Panay Island, the Philippines. Ecosystem Services. 2014;8:128–140.

[26] Gevaña DT, Pulhin FB, Pampolina NM. Carbon stock assessment of a mangrove ecosystem in San Juan, Batangas. Journal of Environmental Science and Management. 2008;11(1).

[27] Phang VX, Chou L, Friess DA. Ecosystem carbon stocks across a tropical intertidal habitat mosaic of mangrove forest, seagrass meadow, mudflat and sandbar. Earth Surface Processes and Landforms. 2015;40(10):1387–1400.

[28] Christensen B. Biomass and primary production of Rhizophora apiculata Bl. in a mangrove in southern Thailand. Aquatic Botany. 1978;4:43–52.

[29] Jachowski NR, Quak MS, Friess DA, Duangnamon D, Webb EL, Ziegler AD. Mangrove biomass estimation in Southwest Thailand using machine learning. Applied Geography. 2013;45:311–321.

[30] Matsui N, Morimune K, Meepol W, Chukwamdee J. Ten year evaluation of carbon stock in mangrove plantation reforested from an abandoned shrimp pond. Forests. 2012;3(2):431–444.

[31] Wilson N. Biomass and regeneration of mangrove vegetation in Kien Giang Province, Vietnam. Deutsche Gesellschaft für Internationale Zusammenarbeit (GTZ); 2010.

[32] Tue NT, Dung LV, Nhuan MT, Omori K. Carbon storage of a tropical mangrove forest in Mui Ca Mau National Park, Vietnam. Catena. 2014;121:119–126.
