# Supplementary material for: The Use of Mixed Effects Models for Obtaining Low-Cost Ecosystem Carbon Stock Estimates in Mangroves of the Asia-Pacific
Source: PLoS One. 2017 Jan 9;12(1):e0169096. doi: 10.1371/journal.pone.0169096 (PMC5222395; doi:10.1371/journal.pone.0169096)
Supplement: S3 File — The best linear unbiased predictors (BLUPs) of the random effects are provided in this file. (PDF) [file pone.0169096.s003.pdf]

Supporting Information 3
Best linear unbiased predictors for the random effect intercept adjustments

Table 1: **Best linear unbiased predictors for the random effect intercept adjustments**

Country	Site	Site code	Biomass value	SOC value
Australia	Dampier	au/da	6.6	11.5
Australia	Bay of Rest	au/br	12.3	11.1
Australia	Mangrove Bay	au/mb	-4.1	17.0
Australia	Port Hedland	au/ph	2.0	6.1
China	Futian Nature Reserve	ch/fnr	12.1	-
China	Leizhou Bay	ch/glb	-5.6	-
F.S. of Micronesia	Kosrae	mi/kos	-2.5	4.7
F.S. of Micronesia	Yap	mi/yap	-3.1	13.7
Indonesia	Bintuni	in/bin	14.0	-3.3
Indonesia	Bunaken	in/bun	22.3	21.3
Indonesia	Kubu Raya	in/kr	14.8	-11.0
Indonesia	Kumai	in/kum	1.7	-12.4
Indonesia	Sembilang	in/sem	27.8	3.6
Indonesia	Sulawesi	in/sul	-21.6	19.3
Indonesia	Tanjung Puting	in/tp	-9.0	-11.0
Indonesia	Teminabuan	in/tem	6.6	-5.8
Indonesia	Timika	in/tim	13.6	11.4
Japan	Manko Wetland	jp/man	7.0	-6.3
Malaysia	Kuala Selangor	ma/ks	3.5	-
Malaysia	Matang	ma/mat	-10.2	-
Malaysia	Sungai Haji Dorani	ma/shd	11.9	-
Myanmar	Ayeyarwady Delta	my/ay	2.1	-
Thailand	Ranong	th/rng	4.9	-
Thailand	Krabi River Estuary	th/kre	-17.6	-9.4
Thailand	Pak Panang	th/pp	-4.2	-15.7
Vietnam	An Bien	vn/ab	-8.4	-8.0
Vietnam	An Minh	vn/am	-7.3	-9.9
Vietnam	Can Gio	vn/cg	-16.2	1.5
Vietnam	Ha Tien	vn/ht	-9.7	-
Vietnam	Hai Phong	vn/hp	-0.2	-7.6
Vietnam	Hon Queo	vn/hq	0.9	-
Vietnam	Kien Luong	vn/kl	-9.5	-6.6
Vietnam	Kien Vang	vn/kv	-15.0	3.1
Vietnam	Mui Ca Mao	vn/mcm	31.5	-10.9
Vietnam	Quang Ninh	vn/qn	13.9	-2.7
Vietnam	Van Rang	vn/vr	-12.6	-9.1
Vietnam	Van Ray	vn/vry	-13.3	-
Vietnam	Vinh Quang	vn/vq	-39.4	-
